# Detection of regional melanoma metastases by ultrasound B-scan, cytology or tyrosinase RT-PCR of fine-needle aspirates

**DOI:** 10.1038/sj.bjc.6690580

**Published:** 1999-07

**Authors:** C Voit, A Schoengen, M Schwürzer, L Weber, T Mayer, T M Proebstle

**Affiliations:** Department of Dermatology, University of Ulm, Ulm, Germany; Department of Medical Oncology, Armed Forces Hospital Ulm, Ulm, Germany; Department of Dermatology of the Charité, Humboldt University Berlin, Germany

**Keywords:** melanoma, ultrasound guided fine needle aspiration cytology (FNAC), tyrosinase reverse transcription polymerase chain reaction (RT-PCR), RT/PCR of fine needle aspiration material (FNA-PCR)

## Abstract

Physical examination and ultrasound B-scan screening are important follow-up procedures in melanoma patients with regional disease. However, they do not allow definite diagnosis of suspicious lesions. Fine-needle aspiration cytology (FNAC) enhances the diagnostic accuracy in such patients but, unfortunately, reaches its technical limits, particularly when very small or necrotic lesions are examined. We therefore tested whether tyrosinase reverse transcription polymerase chain reaction (RT-PCR) of fine-needle aspirates (FNA-PCR) could help to increase diagnostic sensitivity. With clinical follow-up in 69 melanoma patients 81 regional lymph nodes were detected by ultrasound B-scan examination, nine of whom appeared to be palpable. Technically, FNAC was successful in all 81 lymph nodes, while FNA-PCR failed to obtain RNA at detectable levels in two lymph nodes of two patients. Of 79 lesions which have been completely evaluated by B-scan, FNAC and FNA-PCR, 44 proved to be melanoma metastases by histopathology, while the remaining 35 lesions were finally classified as non-specific lymph nodes. Of the 44 melanoma metastases 80% (*n* = 35) have been detected by B-scan, 90% (*n* = 39) by FNAC and 100% (*n* = 44) by FNA-PCR (*P* < 0.05 vs FNAC, *P* < 0.005 vs B-scan). In the subclass of lesions with diameters below 10 mm the sensitivities were 72% (*n* = 13), 78% (*n* = 14) and 100% (*n* = 18) respectively. In 35 regional lymph nodes classified as benign lesions, FNAC was always negative while FNA-PCR produced one positive result. Neither of these methods did produce false positive results in 15 control lymph nodes of non-melanoma patients. We conclude, that FNA-PCR might have superior sensitivity as compared to FNAC or ultrasound B-scan, particularly in melanoma lesions with diameters below 10 mm. © 1999 Cancer Research Campaign


*British Journal of Cancer* (1999) **80**(10), 1672–1677
© 1999 Cancer Research Campaign
Article no. bjoc.1999.0580

### Detection of regional melanoma metastases by ultrasound B-scan, cytology or tyrosinase RT-PCR of fine-needle aspirates

**C Voit[1], A Schoengen[2], M Schwürzer[3], L Weber[1], T Mayer[2] and TM Proebstle[1]**

[1]Department of Dermatology, University of Ulm, Ulm, Germany; [2]Department of Medical Oncology, Armed Forces Hospital Ulm, Ulm, Germany; [3]Department of Dermatology of the Charité, Humboldt University Berlin, Germany

**Summary** Physical examination and ultrasound B-scan screening are important follow-up procedures in melanoma patients with regional disease. However, they do not allow definite diagnosis of suspicious lesions. Fine-needle aspiration cytology (FNAC) enhances the diagnostic accuracy in such patients but, unfortunately, reaches its technical limits, particularly when very small or necrotic lesions are examined. We therefore tested whether tyrosinase reverse transcription polymerase chain reaction (RT-PCR) of fine-needle aspirates (FNA-PCR) could help to increase diagnostic sensitivity. With clinical follow-up in 69 melanoma patients 81 regional lymph nodes were detected by ultrasound B-scan examination, nine of whom appeared to be palpable. Technically, FNAC was successful in all 81 lymph nodes, while FNA-PCR failed to obtain RNA at detectable levels in two lymph nodes of two patients. Of 79 lesions which have been completely evaluated by B-scan, FNAC and FNA-PCR, 44 proved to be melanoma metastases by histopathology, while the remaining 35 lesions were finally classified as non-specific lymph nodes. Of the 44 melanoma metastases 80% ($n$ = 35) have been detected by B-scan, 90% ($n$ = 39) by FNAC and 100% ($n$ = 44) by FNA-PCR ($P$ < 0.05 vs FNAC, $P$ < 0.005 vs B-scan). In the subclass of lesions with diameters below 10 mm the sensitivities were 72% ($n$ = 13), 78% ($n$ = 14) and 100% ($n$ = 18) respectively. In 35 regional lymph nodes classified as benign lesions, FNAC was always negative while FNA-PCR produced one positive result. Neither of these methods did produce false positive results in 15 control lymph nodes of non-melanoma patients. We conclude, that FNA-PCR might have superior sensitivity as compared to FNAC or ultrasound B-scan, particularly in melanoma lesions with diameters below 10 mm.

**Keywords:** melanoma; ultrasound guided fine needle aspiration cytology (FNAC); tyrosinase reverse transcription polymerase chain reaction (RT-PCR); RT/PCR of fine needle aspiration material (FNA-PCR)

Melanoma follow-up programmes in our department include clinical investigation and ultrasound B-scans of regional lymph node basins at 3-month intervals for all patients with high risk primary tumours. If subcutaneous (s.c.) lesions or suspicious lymph nodes are detected, fine-needle aspirates are obtained for cytological examination (FNAC) (Perry et al, 1988; Fornage and Lorigan, 1989; Schoengen et al, 1993; Basler et al, 1997). Routine use of FNAC allows definite diagnosis of melanoma metastases within less than 1 day, it speeds up the organization of necessary staging procedures, spares the patient from diagnostic surgery and therefore is also a cost-decreasing approach. However, in necrotic or small lesions with diameters less than 10 mm, FNAC frequently reaches its technical limits, especially in those cases, when fine-needle aspiration comprises less than 100 cells for each smear. Detection of tyrosinase-RNA, a tissue-specific enzyme regulating melanin biosynthesis (Kwon et al, 1987; Ponnazhagen et al, 1994), by use of reverse transcription (RT) and polymerase chain reaction (PCR) was originally described for melanoma cells circulating in peripheral blood (Smith et al, 1991). The use of the tyrosinase-RT-PCR method was later transferred to detect occult melanoma micrometastases in lymph nodes (Wang et al, 1994; Schwürzer-Voit et al, 1996; van-der-Verde et al, 1996) and also in the s.c. fat tissue adjacent to primary melanomas (Proebstle et al, 1996).

The aim of the present study was to evaluate the sensitivity of the tyrosinase RT-PCR in fine-needle aspirates (FNA-PCR) in conjunction with ultrasound B-scan and FNAC examination with particular regard to putative metastases of small size with diameters less than 10 mm.

*Received 9 September 1998*
*Revised 21 January 1999*
*Accepted 27 January 1999*

*Correspondence to:* T Mayer, Abteilung Innere Medizin/Onkologie, Bundeswehrkrankenhaus Ulm, Oberer Eselsberg 40, 89081 Ulm, Germany

### MATERIALS AND METHODS

### Patients

Study patients were recruited from our clinical follow-up programme for melanoma patients. Sixty-nine patients who had presented before without regional disease developed 81 new regional lesions which were worked up by the use of different diagnostic tools as described in the paper. The outcome of diagnostic procedures showed that of these 69 patients 34 had a regional recurrence of melanoma, 24 of whom were in clinical stage III and ten in stage IV (AJCC). Additionally, 33 disease-free melanoma patients and two stage IV patients did not have a regional lymph node recurrence. Concomitantly, 15 patients with non-melanoma disease underwent the same diagnostic programme for control purposes (Table 1).

Patients were seen for clinical follow-up at 3-monthly intervals. All patients received a thoroughly performed inspection and

**Table 1** Patients' characteristics

| | Melanoma patients | | Non-melanoma patients | |
|---|---|---|---|---|
| | Metastatic | Disease-free | Other malignancies[a] | Benign diagnosis |
| Number | 34 | 35 | 9 | 6 |
| Female sex | 53% | 66% | 67% | 100% |
| Age, median (range) | 52 (24–87) | 52 (25–83) | 72 (67–86) | 39 (18–56) |

[a]Diagnoses were non-Hodgkin's lymphoma (*n* = 6), malignant fibrous histiocytoma (*n* = 1), malignant schwannoma (*n* = 1) and thyroid carcinoma (*n* = 1).

**Table 2** Morphological criteria for evaluation of lymph nodes and melanoma metastases by ultrasound B-scan

| | |
|---|---|
| Non-specific lymph node | Central ultrasound reflexes, reflex-free margin, ellipsoid shape |
| No clear diagnosis possible | Uncertain reflex pattern, uncertain shape, diameter below 5 mm |
| Melanoma metastasis | Whole lesion reflex-free, spherical or balloon-like shape |

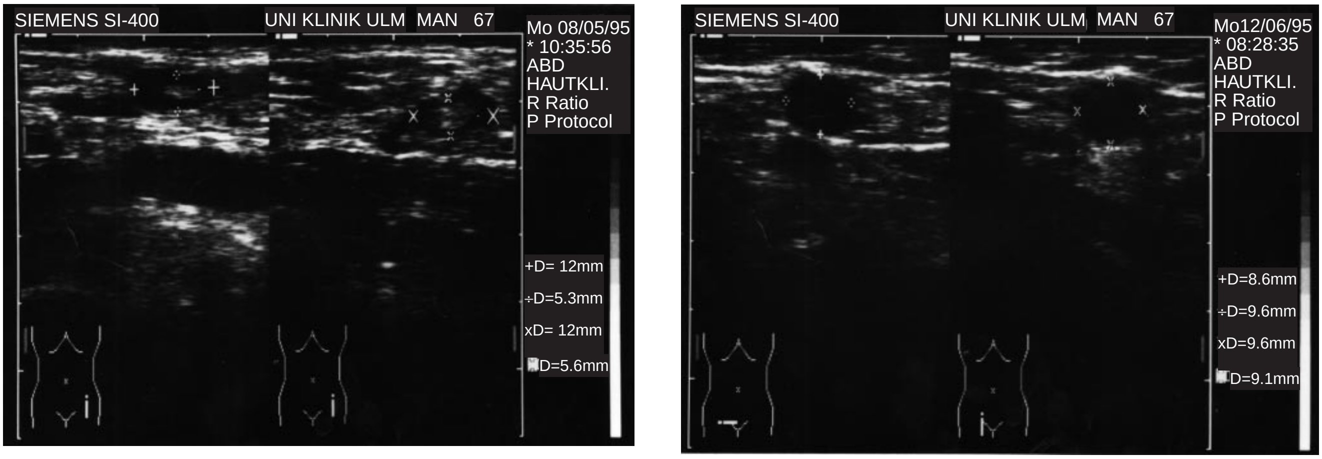

**Figure 1** (**A**) Cross-sections of a non-specific lymph node. Ultrasound B-scan shows an ellipsoid lesion with central reflexes and a reflex-free margin in the left groin of a 37-year-old patient with stage IV melanoma. FNA-PCR and FNAC were negative. (**B**) Ultrasound B-scan cross-section showing a balloon-shaped, reflex-free tumour in the right groin of a 34-year-old patient with a primary melanoma originally located at her right lower leg. FNAC, FNA-PCR and histopathology revealed a melanoma metastasis physical examination of the former site of the primary tumour, the regional lymph node basins, the in-transit distance as well as a control of the whole integument. The study was reviewed and approved by the local ethical committee and written consent was obtained from all patients.

### Ultrasound B-scan

An ultrasound (US) B-scan with a 7.5 MHz linear scanner (Sonoline, Siemens, Munich, Germany) was used throughout the study. Sonographic criteria of lymph nodes and metastatic tumours were applied as listed in Table 2 to reduce operator dependent interpretation to a minimum. Figure 1 illustrates an example for a non-specific lymph node with regular architecture and typical echo-pattern (Figure 1A) and an example for a regional lymph node metastasis (Figure 1B).

### FNAC

Fine needle aspiration biopsies were performed as described (Schoengen et al, 1993). To ensure appropriate tissue sampling the aspiration procedure was guided by ultrasound B-scan to control the precise location of the needle tip. Incidental aspiration of tissue is avoided by applying suction exclusively at the site of interest. The aspirate was then spread on a glass slide and stained by May–Grünwald–Giemsa to allow cytological analysis (Figure 2).

### Tyrosinase FNA-PCR

About 0.3 µl of aspirate were immediately shock-frozen in liquid nitrogen and stored at –80°C for subsequent molecular biological evaluation. Total RNA was extracted from the mini-cell-pellet by means of Rneasy TM total RNA kit® (Qiagen, Hilden, Germany). RNA is extracted from homogenized cell material via binding to a silica gel membrane. All precaution was taken to ensure integrity and purity of RNA. RNA was quantified by UV spectrophotometry at 260 nm and 280 nm and stored at –80°C. A total of 1.5 µg of total RNA was transcribed by means of 50 ng random hexamers, 10 mM dNTP-mix and 200 units of superscript II RT (Gibco, BRL, Grand Island, NY, USA). First the mixture was incubated for 50 min at 42°C, then heated up to 70°C to stop RT. After cooling on ice 2 U $\mu l^{-1}$ *Escherichia coli* RNAase H (Gibco BRL) was added at a temperature of 37°C. Twenty minutes later the obtained cDNA was stored at –20°C for subsequent investigation. cDNA of the SK-mel-28 melanoma cell line (American Type Culture Collection) served as positive control. Primers for a tyrosinase PCR designed as a nested PCR were used as described (Smith et al, 1991). The outer primers HTYR 1 (TTGGCAGATTGT-CTGTAGCC) and HTYR 2 (AGGCATTGTGCATGCTGCTT)

© 1999 Cancer Research Campaign

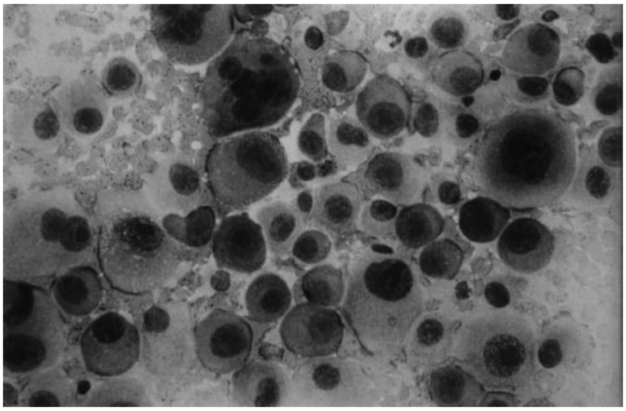

**Figure 2** FNAC of a melanoma metastasis presenting with large, dissociated, well conserved pleomorphic cells with large nuclei, high nucleus plasma relation, atypical mitoses and megacaryotypic cells. Epithelial-cell-like type of melanoma cells. May–Grünwald–Giemsa-stain amplify a product of 284 base pairs, while the inner primers HTYR 3 (GTCTTTATGCAATGGAACGC) and HTYR 4 (GCTATCCCAGTAAGTGGACT) amplify a product of 207 base pairs. Two microlitres of cDNA from lymph node aspirates, blood samples of 1 µl of cDNA of SK-mel 28 melanoma cells were added to 45 µl PCR starting mixture. Each sample was overlaid with two drops of mineral oil to prevent evaporation. First delay: 3 min at 94°C, then first hold at 80°C and addition of 1 unit *Taq* DNA polymerase (Gibco BRL). Thirty cycles were performed, each consisting of 45 s at 94°C, 45 s at 60°C and 45 s at 72°C. Last delay: 5 min at 72°C and finally at 30°C. Five microlitres of the first PCR-product diluted 1:100 were used for the nested PCR. The nested PCR was carried out in the same way as the first PCR. To avoid contamination the reaction mixtures were performed in a fume hood. Ten microlitres of the second amplified product were analysed on a 3% agarose gel followed by ethidiumbromide staining (ca. 80 min, 80 V). One hundred base pair ladder (Gibco BRL) was used as standard. PCR samples free of cDNA served as negative controls, PCR samples with cDNA of SK-mel-28 melanoma cell line served as positive control. SK-mel-28 cells were cultured in RPMI-1640 with 10% heat inactivated fetal calf serum (Gibco BRL) plus penicillin, streptomycin, 1% L-glutamine and 1% non-essential amino acids. Negative and positive controls are run on each agarose gel in addition to patients' samples. To ensure that RNA in the tyrosinase gene negative samples has not been degraded, a parallel PCR run for the detection of glyceraldehyde 3-phosphatase dehydrogenase (GAPDH) (housekeeping enzyme) was performed. The specificity of PCR-products was examined by sequencing. cDNA was amplified by using HTYR 3/4 with previously marked universal primers M13 (forward and reverse). Sequencing was performed via dye primer sequencing (Perkin-Elmer). The analysis of each specimen was carried out on a 373 DNA sequencer (ABI, Applied Biosystems).

### Tyrosinase RT-PCR from peripheral blood

Blood samples from patients were taken at the same time as fine-needle aspiration biopsies were performed. For RNA preparation, 5 ml EDTA blood were treated with erythrocyte lysis buffer. Extraction of total RNA was performed by means of Qia shredder columns and Rneasy TM total RNA kit® (Qiagen GMBH, Hilden,

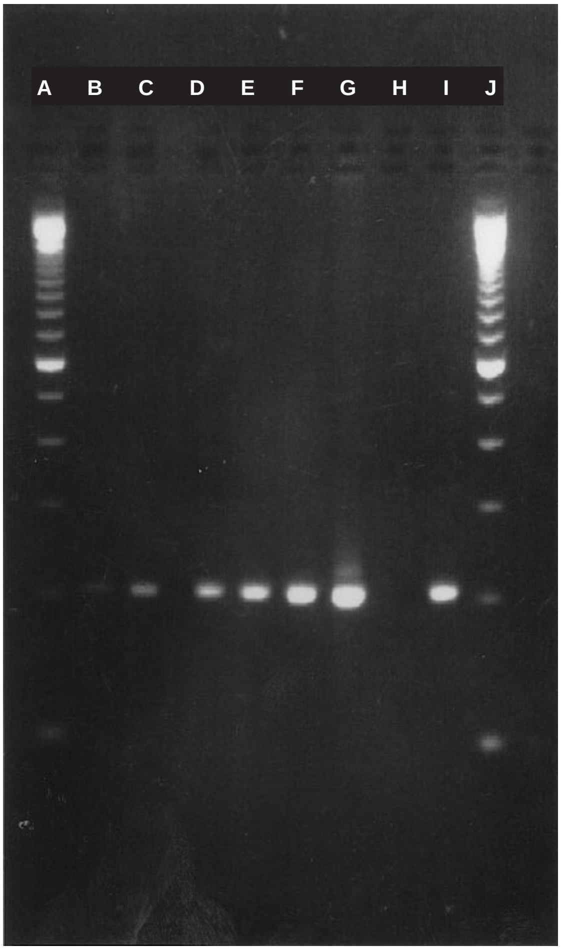

**Figure 3** Detection of tyrosinase-specific RNA in a sensitivity assay. Five millilitres of blood of healthy donors was mixed with 1 up to $10^6$ SK-mel-28 cells. Lane A, J: 100 bp ladder; lanes B–G: serial dilutions with 1, 10, $10^2$, $10^3$, $10^4$, $10^5$ and $10^6$ cells; lane H: negative control; lane I: positive control

Germany). A total of 1.5 µg of RNA were used for synthesis of cDNA. Each PCR course was controlled by a second run starting from RNA preparation to cDNA synthesis and PCR procedure to confirm results. PCR from RNA as template, i.e. PCR without RT was carried out to exclude amplification of genomic cDNA, which would lead to false-positive results. Processing blood shortly after collection was regarded as being of great importance.

Tenfold serial dilutions of SK-mel 28 melanoma cells in peripheral blood from a healthy donor were performed. One SK-mel-28 melanoma cell in a background of $10^6$ peripheral blood mononuclear cells could be detected (Figure 3).

### Statistics

McNemar's test was used to detect significant differences in the sensitivity of diagnostic procedures. *P*-values were given one-sided throughout the paper.

### RESULTS

Eighty-one regional lesions were detected with routine clinical follow-up in 69 patients with malignant melanoma by ultrasound

 © 1999 Cancer Research Campaign

**Table 3** Histopathologically proven regional melanoma metastasis which had been investigated before by ultrasound B-scan, FNAC and FNA-PCR

| | Tumours < 10 mm (12 patients) | Tumours > 10 mm (22 patients) | Total (34 patients) |
|---|---|---|---|
| Physical examination | $n = 1$ (6%) $P < 0.0001$ | $n = 8$ (31%) $P < 0.0001$ | $n = 9$ (20%) $P < 0.0001$ |
| Ultrasound B-scan | $n = 13$ (72%) $P < 0.05$ | $n = 22$ (85%) $P < 0.07$ | $n = 35$ (80%) $P < 0.005$ |
| FNAC | $n = 14$ (78%) $P < 0.07$ | $n = 25$ (96%) $P = 0.5$ | $n = 39$ (89%) $P < 0.05$ |
| Diagnosed by FNA-PCR | $n = 18$ (100%) | $n = 26$ (100%) | $n = 44$ (100%) |
| Total number of tumours examined | $n = 18$ (100%) | $n = 26$ (100%) | $n = 44$ (100%) |

*P*-values are given in comparison to the FNA-PCR result of the same column. The number *n* denotes the number of lesions.

**Table 4** Number of relapses and time interval to relapse of disease-free melanoma patients, calculated from the time of their FNAC and FNA-PCR investigation of regional lymph nodes

| Clinical stage (AJCC) | I | II | III | IV | Total |
|---|---|---|---|---|---|
| Number of patients | 18 | 10 | 5 | 2 | 35 |
| Number of relapses | 0 | 2 | 2 | NA | 4 |
| Intervals to relapse (months) | – | 14, 15[a] | 10, 12 | NA | – |
| Interval of follow-up since FNAC in months as median (range) | 30 (15–39) | 29.5 (9–32) | 34 (27–39) | NA | 31.5 (9–39) |

[a]NA = not applicable, because of lack of disease-free interval in stage IV patients. Relapse at the site tested by FNAC and FNA-PCR 15 months later.

B-scan examination, nine lesions appeared to be palpable. At the same visit, FNAC and tyrosinase FNA-PCR examination were performed, but inadequate handling caused loss of RNA in two samples of two patients. Therefore, material obtained from 79 lesions of 67 patients was left for comparison of sensitivities. Forty-four lesions were histopathologically proved to be regional melanoma metastases of 34 patients (Table 1). Another 35 disease-free melanoma patients had 35 non-specific regional lymph nodes as shown by repeated control with clinical follow-up. Furthermore, 15 lymph nodes of 15 non-melanoma patients were examined in the same fashion.

### Melanoma metastases

Before surgical removal for histopathological examination, 44 regional or in-transit melanoma metastases derived from 34 patients had been simultaneously examined by ultrasound B-scan, FNAC and by FNA-PCR (Table 3). Only 20% of these lesions ($n = 9$, $P < 0.0001$) had been detected as palpable tumours by physical examination, whereas 80% of these tumours ($n = 35$, $P < 0.005$) were classified as melanoma metastases by ultrasound B-scan. Furthermore, 89% ($n = 39$, $P < 0.05$) of these metastases were diagnosed by FNAC and, finally, tyrosinase FNA-PCR was able to recognize all malignant lesions ($n = 44$). The *P*-values indicate significantly reduced sensitivities for all other methods if compared to FNA-PCR.

In the subclass of lesions with diameters less than 10 mm, only one lesion (6%, $P < 0.0001$) was palpable, ultrasound was able to detect 72% ($n = 13$, $P < 0.05$), FNAC diagnosed 78% ($n = 14$, $P < 0.07$), whereas FNA-PCR again identified 100% ($n = 18$) of the suspicious lesions as melanoma metastases. Among lesions larger than 10 mm in diameter, physical examination detected 31% ($n = 8$, $P < 0.0001$), ultrasound B-scan 85% ($n = 22$, $P < 0.07$), FNAC 96% ($n = 25$, $P = 0.5$) and FNA-PCR again 100% of the melanoma metastases.

**Table 5** Percentage of melanoma patients with positive tyrosinase RT-PCR from peripheral blood at time of fine-needle aspiration procedure

| Clinical stage (AJCC) | I | II | III | IV | All stages |
|---|---|---|---|---|---|
| Positive patients with metastatic disease | – | – | 47% (9/19) | 70% (7/10) | 55% (16/29) |
| Positive patients with disease-free melanoma | 0% (0/18) | 20% (2/10) | 40% (2/5) | 100% (2/2) | 17% (6/35) |
| All melanoma patients | 0% (0/18) | 20% (2/10) | 46% (11/24) | 75% (9/12) | 34% (22/64) |

Total numbers are given in brackets.

### Regional lymph nodes of disease-free melanoma and non-melanoma patients

Thirty-five non-specific regional lymph nodes of disease-free melanoma patients, nine lymph nodes of patients with other malignancies and six lymph nodes of patients with benign diagnoses were investigated in the same way as described above for melanoma metastases (Table 1). A typical lymph node architecture as confirmed by ultrasound B-scan was necessary to be included in this group. FNAC revealed negative results in each of these lymph nodes. Likewise, FNA-PCR was clearly negative in all non-melanoma patients. However, one lymph node of a disease-free melanoma patient was positive by RT-PCR but did not relapse until now during a 24-month period. The RT-PCR for tyrosinase in the peripheral blood of this patient was negative at the time of FNA-PCR.

The median follow-up period in the group of disease-free melanoma patients was at the time of writing this paper 31.5 months (range 9–39 months) (Table 4). Until now, only four relapses have been observed in this group, all in patients with clinical stages II and III (AJCC). However, only one of these relapses originated in a lymph node basin which was examined before by ultrasound B-scan, FNAC or RT-PCR. The interval between study examination and melanoma relapse was 15 months in this case.

### Tyrosinase RT-PCR from peripheral blood

In addition, we performed tyrosinase-RT-PCR from peripheral blood of melanoma patients and of patients with non-melanoma disease. RT-PCR from blood of non-melanoma patients was exclusively negative. Table 5 shows the PCR results for all melanoma patients, from whom a peripheral blood sample was obtained at

*© 1999 Cancer Research Campaign* 

time of fine-needle aspiration. Patients who presented with melanoma metastases were positive for tyrosinase RT-PCR from peripheral blood in 47% in clinical stage III, and in 70% in stage IV disease respectively. Disease-free melanoma patients who underwent fine-needle aspiration from non-specific lymph nodes exhibited also a stage dependency of positive PCR results when peripheral blood was tested. Examining blood samples, tyrosinase transcripts were detected in none of 18 patients in clinical stage I; however, in two out of ten patients in stage II, in two out of five in stage III and in two out of two patients in stage IV.

### DISCUSSION

Physical examination at short time intervals is the basis in follow-up regimens of high risk melanoma patients. Despite a lack of definitive criteria, during the past decade ultrasound B-scan examination of soft tissues has become an additional powerful tool, particularly for detection of subcutaneous in transit lesions and regional lymph node metastases. Meanwhile, in our department, only 20% of lymph node metastases in melanoma patients are diagnosed by physical examination, whereas 80% are identified by ultrasound B-scan (Table 3). Routine evaluation of these lesions by FNAC has accelerated and simplified the planning of both, staging procedures and therapeutic measures. Diagnostic surgery, therefore, has become an extraordinarily rare event. Unfortunately, the successful use of FNAC needs to meet two major criteria. It first has to be performed by well-trained experts and, second, the risk of producing low quality smears with low cell numbers is increased in the case of smaller lesions. Until now, most studies focusing on the FNAC technique reported exceedingly high rates for sensitivity and specificity (Perry et al, 1988; Fornage and Lorigan, 1989; Schoengen et al, 1993; Basler et al, 1997), yet were based on results obtained from aspirates of larger tumours and, originated from clinical centres where experts investigate large numbers of FNAC samples each year. One aim of this study was to find out whether the FNA-PCR method could reproduce these results. This would be important, because RT-PCR analysis can be performed by technical assistance staff at low cost once a PCR laboratory is established.

Since detection of circulating melanoma cells using an RT-PCR method for identification of tyrosinase transcripts in peripheral blood (Smith et al, 1991), despite the extraordinary capability to detect one melanoma cell in 10 ml of blood (Brossart et al, 1993), various studies differ in their interpretation with regard to stage dependency and prognostic meaning of such a finding (Brossart et al, 1993; Hoon et al, 1995; Kunter et al, 1996; Mellado et al, 1996; Pitman et al, 1996). However, undoubtedly, tyrosinase, a rather tissue specific enzyme, should be of principal use for detection of melanoma cells also in other tissues but blood. In-situ hybridization experiments in mice demonstrated that expression of the tyrosinase gene is restricted to melanocytes in skin, hair follicles and uvea (Beermann et al, 1992). In Northern blot analysis of mouse RNA, tyrosinase transcription could be demonstrated in melanocytes and to a lesser extent in normal testes, whereas brain, lung, heart, kidneys, liver and muscles were negative (Muller et al, 1988).

At present, only a few papers address the use of tyrosinase RT-PCR for detection of melanoma cells in surgically removed lymph nodes (Wang et al, 1994; Van-der-Velde et al, 1996) or in fine-needle aspirates derived from putative lymph node or in transit metastases (Schwürzer-Voit et al, 1996). Studies to evaluate this method in the context of routine clinical patient management are urgently needed. Our study indicates that tyrosinase RT-PCR could be of use in diagnosis of melanoma metastases. In case of a positive RT-PCR, in our clinic definitive tumour surgery is warranted directly, instead of open node biopsy. On the other hand, in case of a negative RT-PCR from an ultrasonographically suspicious lesion, control by B-scan examination and FNA-PCR in short intervals would allow to recognize a slow growing metastasis still in time. Therefore, FNA-PCR together with B-scan examination can properly fit into already published diagnostic algorithms of unclear lesions (Basler et al, 1997), and may help to make early diagnosis of metastatic disease less invasive and less related to severe morbidity.

None of the lesions of control patients with inflammatory disease or non-melanoma malignancy was positive when examined by FNA-PCR technique. Only one lymph node shown to be non-specific in architecture by ultrasound B-scan was positive by FNA-PCR without evidence of malignant disease now for 24+ months. Evaluating clinically and ultrasonographically suspicious or unclear lesions, which subsequently were diagnosed as melanoma metastases on histological grounds, the FNA-PCR procedure was shown to exceed the sensitivity of the classic FNAC method. This was particularly true for lesions smaller than 10 mm in diameter. However, with lesions larger than 10 mm of diameter we were not able to show a significantly increased sensitivity of FNA-PCR as compared to ultrasound B-scan ($P < 0.07$) or FNAC ($P = 0.5$) alone.

One major point of criticism against RT-PCR of fine-needle aspirates would be the argument of possible blood contamination of aspirates, since circulating melanoma cells may influence the result of the FNA-PCR. We therefore analysed blood samples, obtained simultaneously with fine-needle aspirates, for the presence of tyrosinase transcripts. As shown in Table 5, the rate of positive blood samples does not differ significantly for clinical stages III or IV disease, regardless, whether they had a melanoma metastasis or whether they were in a disease-free state at the time of FNA-PCR. Furthermore, in only one patient who had a negative blood test for tyrosinase, FNA-PCR of non-specific lymph nodes in melanoma patients had negative results despite the fact that 17% of those patients exhibited positive blood samples.

### CONCLUSION

We conclude that FNA-PCR examination of ultrasonographically suspicious or unclear tumours might be of value in clinical management of melanoma patients. In cases of large metastases with diameters over 10 mm it facilitates the diagnosis if the expertise of a well-trained cytologist is not available. However, for the diagnosis of metastases with diameters less than 10 mm, FNA-PCR proves to be highly sensitive and even superior to FNAC. To determine its role in sentinel node staging should be of great interest.

### ACKNOWLEDGEMENTS

Special thanks to Mrs Antje Wunderlich for expert technical assistance and to Prof. Wolfram Sterry and Prof. Jürgen Knop for highly appreciated comments.

### REFERENCES

Basler GD, Fader DJ, Yahanda A, Sondak VK and Johnson TM (1997) The utility of fine needle aspirations in the diagnosis of melanoma metastatic to lymph nodes. *J Am Acad Dermatol* **36**: 403–408

© 1999 Cancer Research Campaign

Beermann F, Schmid E and Schutz G (1992) Expression of the mouse tyrosinase gene during embryonic development: recapitulation of the temporal regulation in transgenic mice. *Proc Natl Acad Sci USA* **89**: 2809–2813

Brossart P, Keilholz U, Willhauck M, Scheibenbogen C, Möhler T and Hunstein W (1993) Hematogenous spread of malignant melanoma cells in different stages of disease. *J Invest Dermatol* **101**: 887–889

Fornage BD and Lorigan JG (1989) Sonographic detection and fine-needle aspiration biopsy of non-palpable recurrent of metastatic melanoma in subcutaneous fatty tissues. *J Ultrasound Med* **8**: 421–424

Hoon DSB, Wang Y, Dale PS, Conrad AJ, Garrison D, Kuo C, Foshag LJ, Nizze AJ and Morton DL (1995) Detection of occult melanoma cells in blood with a multiple-marker polymerase chain reaction assay. *J Clin Oncol* **13**: 2109–2115

Jung FA, Buzaid AC, Merrick IR, Ross MI, Woods KV, Lee JJ, Albitar M and Grimm EA (1997) Evaluation of tyrosinase mRNA as a tumor marker in the blood of melanoma patients. *J Clin Oncol* **15**: 2826–2831

Kunter U, Buer J, Probst M, Densing S, Dallmann I, Grosse J, Kirchner H, Schluepen EM, Volkenandt M, Ganser A and Atzpodien J (1996) Peripheral blood tyrosinase messenger RNA detection in malignant melanoma. *J Natl Cancer Inst* **88**: 569–570

Kwon BS, Haq AK, Pomerantz SH and Holaban R (1987) Isolation and sequence of a cDNA clone for human tyrosinase that maps at the mouse c-albino locus. *Proc Natl Acad Sci USA* **84**: 7473–7477

Mellado B, Colomer D, Castel T, Munoz M, Carballo E, Galan M, Mascaro JM, Vives-Corrons JL, Grau JJ and Estape J (1996) Detection of circulating neoplastic cells by reverse transcriptase polymerase chain reaction in malignant melanoma: association with clinical stage and prognosis. *J Clin Oncol* **14**: 2091–2097

Muller G, Ruppert S, Schmid S and Schulz G (1988) Functional analysis of alternatively spliced tyrosinase gene transcripts. *EMBO J* **7**: 2723–2730

Perry DP, Seigler HF and Johnston MW (1988) Diagnosis of metastatic malignant melanoma by fine needle aspiration biopsy: a clinical and pathologic correlation of 298 cases. *J Natl Cancer Inst* **77**: 1013–1021

Pitman K, Burchill S, Smith B, Southgate J, Gore M and Selby P (1996) Reverse transscriptase polymerase chain reaction for expression of tyrosinase to identify malignant melanoma cells in peripheral blood. *Ann Oncol* **7**: 197–201

Ponnazhagen S, Hou L and Kwon BS (1994) Structural organization of the human tyrosinase gene and sequence analysis and characterization of its promoter region. *J Invest Dermatol* **102**: 744–748

Proebstle T, Huber R and Sterry W (1996) Detection of early micrometastases in subcutaneous fat of primary malignant melanoma patients by identification of tyrosinase mRNA. *Eur J Cancer* **32A**: 1664–1667

Reinhold U, Lüdke-Handjery HC, Schnautz S, Kreysel HW and Abken H (1997) The analysis of tyrosinase-specific mRNA in blood samples of melanoma patients by RT-PCR is not a useful test for metastatic tumor progression. *J Invest Dermatol* **108**: 166–169

Schoengen A, Binder T, Faiss S, Weber L and Zeelen U (1993) Feinnadelaspirations-zytologie beim metastasierenden malignen Melanom: Verbesserung der Ergebnisse durch Ultraschallführung. *Hautarzt* **44**: 703–707

Schwürzer-Voit M, Proebstle TM and Sterry W (1996) Identification of lymph node metastases by use of PCR in melanoma patients. *Eur J Cancer* **32A**: 264–268

Smith B, Selby P, Southgate J, Pitman K, Bradley C and Blair CE (1991) Detection of melanoma cells in peripheral blood by means of reverse transcriptase and polymerase chain reaction. *Lancet* **338**: 1227–1229

Van-der-Velde-Zimmermann D, Roijers JF, Bouwens-Rombuds A, De-Weger RA, DeGraaf PW, Tilanus MG and Van-den-Tweel JG (1996) Molecular test for the detection of tumor cells in blood and sentinel nodes. *Am J Pathol* **149**: 759–764

Wang X, Heller R, VanVoorhis N, Cruse CW, Glaas F, Fenske N, Berman C, Messina JL, Rappaport D, Wels K, DeConti R, Moscinski L, Stankard C, Pulea C and Reintgen D (1994) Detection of submicroscopic lymph node metastases with polymerase chain reaction in patients with malignant melanoma. *Ann Surg* **220**: 768–774

*© 1999 Cancer Research Campaign*
